# An unusual cause of spontaneous bleeding in the intensive care unit – mastocytosis: a case report

**DOI:** 10.1186/1757-1626-1-100

**Published:** 2008-08-18

**Authors:** Martial Koenig, Jérôme Morel, Jacqueline Reynaud, Cécile Varvat, Pascal Cathébras

**Affiliations:** 1 Department of internal medicine, Centre Hospitalier Universitaire de Saint-Etienne, F-42055 Cedex 2, France; 2 Department of intensive care, Centre Hospitalier Universitaire de Saint-Etienne, F-42055 Cedex 2, France; 3 Department of haematology, Centre Hospitalier Universitaire de Saint-Etienne, F-42055 Cedex 2, France

## Abstract

We report the case of a 39-year old male patient who presented with anaphylactoid shock and diffuse bleeding with prolonged activated partial thromboplastin time at the emergency room. The diagnosis of aggressive mastocytosis was suspected and then confirmed by raised tryptase level and mastocytic infiltration of the bone marrow. The outcome was favorable with supportive measures, antihistamine drugs, and imatinib mesylate.

## Background

Mastocytosis is a rare and heterogeneous disease characterized by the presence of excessive numbers of mast cells in various organs, mainly the skin and the bone marrow [1]. Systemic symptoms are related to mast cell degranulation. Although heparin is one of the mediators released by mast cells, spontaneous bleeding has been very rarely reported in mastocytosis.

## Case presentation

**Figure 1:**
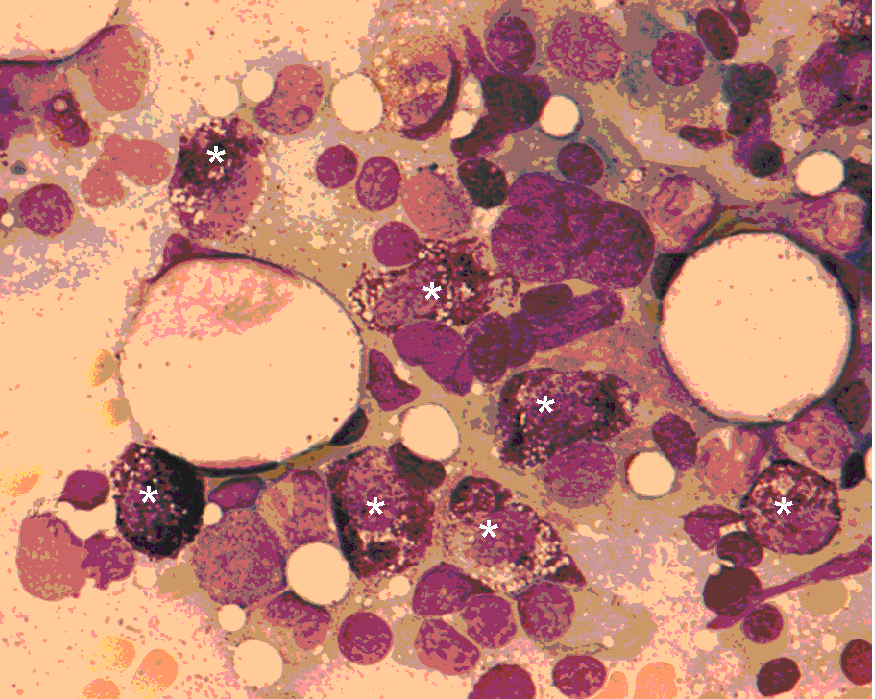
Bone marrow aspiration showing infiltration with mast cells (*).

On December 2006 21^th^, a 39-year-old male patient of North-African origin, non smoker and who did not drink alcohol, with unremarkable family and personal medical history, at the exception of a history of skin rash after intake of aspirin, presented to the emergency room of a general hospital with abdominal pain, vomiting, and a diffuse skin rash. The patient was alert and afebrile. There was no abdominal tenderness. Initially, blood pressure was 170/90 mm Hg and pulse rate was 70/mn, but hemodynamic parameters rapidly deteriorated despite fluid infusion. Laboratory investigations revealed acute renal failure (creatinine 211 μmol/L), hypokaliemia (2.6 mmol/l), hemoconcentration (protidemia 92 g/L), and clotting tests showed prolonged activated partial thromboplastin time (aPTT) (187" *versus *30" control value) and prothrombin time (17"2 *versus *11"8 control value). Blood count showed hyperleucocytosis (18 × 10^9^/L) mainly due to a rise in neutrophil count (16 × 10^9^/L) with a normal platelet and eosinophil count. Haemoglobin level was 12.6 g/dL. C-reactive protein was only slightly elevated (14 mg/L). The patient was transferred to the intensive care unit of the university hospital because of anuria and unexplained abnormalities of clotting tests. On admission, diffuse skin rash was still present. The patient presented signs of shock (blood pressure 80/60 mm Hg, pulse rate 130/mn, anuria and agitation). Orotracheal intubation was thus performed, and mechanical ventilation and continuous hemofiltration were started. Epinephrine infusion corrected hemodynamic status, and the skin rash quickly disappeared. Septic or toxic shock were the first hypotheses investigated, but no infection was documented, and there was no argument for disseminated intravascular coagulation, since the platelet count remained normal. Repeated clotting tests showed however an aPTT up to 200", a prothrombin time raised up to 90", and anti-Xa activity was 2.5 UI/mL. Fibrinogen was 1.8 g/L, antithrombin level was 56%. These abnormalities persisted despite the infusion of 10 units of fresh frozen plasma, and haemoglobin level dropped to 8.6 g/dl because of diffuse bleeding at the sites of venous puncture. Total body computed tomography showed no cerebral haemorrhage, no organomegaly, but an hematoma of the duodenal wall was noticed. Gastroscopy revealed non-specific oesophagitis without active bleeding. Medical records revealed that the patient had not received any anticoagulant treatment prior or since he was admitted to the hospital, and thus the abnormalities of clotting tests were attributed to an endogenous heparin-like factor production. This hypothesis, combined with initial symptoms of vasoplegic shock, led the hemostasis specialist to suggest to the clinicians the diagnosis of systemic mastocytosis, which was confirmed by subsequent workup, including a serum tryptase level up to 200 μg/L (normal < 13) and a bone marrow biopsy showing multifocal infiltrates of spindle-shaped mast cells [Figure 1]. The patient was initially treated with fresh frozen plasma and red cell transfusions, and then protamine was infused at a rate of 1200 UI/h combined with IV glucocorticoids, enteral H_1 _and H_2 _antihistamines, and imatinib mesylate (400 mg/d). aPTT and prothrombin time were normalized within four days. The patient's status allowed his discharge from intensive care unit after 15 days. On clinical examination in the internal medicine unit, urticaria pigmentosa with Darier's sign (urtication reaction at the site of the papulo-macular lesions when scratched) was demonstrated on the trunk. The patient had not noticed these reddish-brown spots before. Complementary workup revealed long bones involvement on radiographics, diffuse bone abnormality on technetium scintography, diminished bone mineral density (lumbar T-score -1.4; femoral T-score -0.8), and the presence of mastocytic infiltrates in oesophageal wall. No skin biopsy was performed. *C-kit *mutation D816V was not demonstrated. Serum tryptase level was 13.5 μg/L (N < 13) on day 15. A final diagnosis of aggressive systemic mastocytosis was established, and the patient was discharged on day 30 with ranitidine, cetirizine, glucocorticoids, alendronate, and imatinib mesylate (200 mg/d). On his last follow-up visit in June 2008, he remained asymptomatic under the same treatment at the exception of steroids which had been discontinued.

## Conclusion

Systemic mastocytosis is a rare disease characterized by abnormal growth and accumulation of mast cells in various organ [1]. It can follow a benign or indolent course, or it may be associated with invalidating or even life-threatening symptoms such as hypotension, syncope, flushing, urticaria, bronchospasm, peptic ulcer disease, diarrhea, malabsorption, osteoporosis, weigh loss and fatigue [1]. Patients with aggressive systemic mastocytosis usually present with enlarged liver, spleen, and lymph nodes, with or without eosinophilia [1]. The diagnosis of systemic mastocytosis is established by demonstrating mast cell infiltration in an involved tissue, particularly the bone marrow, using special staining techniques or flow cytometry, but the measurement of serum tryptase is a good screening test, since almost all patients with systemic mastocytosis have serum tryptase levels exceeding 20 ng/mL [2]. Clinical pattern depends on mast cells burden in different organs and release of clinically relevant mediators such as histamine, leukotrienes, tryptase and heparin [1,2]. Kinetics of blood clotting may be altered due to fibrinogenolytic and anticoagulant activities of tryptase and heparin respectively [3]. Severe bleeding leading to the death of a patient with systemic mastocytosis due to heparin-like anticoagulant has been recently reported [4], and may represent a difficult diagnosis and a therapeutic challenge in the emergency room. The treatment of systemic mastocytosis is mainly focused on avoidance of triggering factors (e.g. physical stimuli such as heat or cold, alcohol, drugs such as aspirin and other NSAIDS) and symptomatic therapy (H1 and H2 antihistamines, proton pump inhibitors, antileukotrienes, anticholinergics, glucocorticoïds, and epinephrine in case of systemic hypotension). In aggressive forms of systemic mastocytosis, treatments such as interferon alpha, cladribin, and imatinib mesylate should to be considered. Imatinib seems to be more effective in patients without the D816V C-kit mutation [2].

## Abbreviations

aPTT: activated partial thromboplastin time.

## Competing interests

The authors declare that they have no competing interests.

## Authors' contributions

MK took care of the patient in the internal medicine department and wrote the first version of the manuscript. PC took care of the patient in the internal medicine department, revised and edited the manuscript, and is the attending physician of the patient. JR performed the clotting assays and suggested the diagnosis of mastocytosis to the clinicians. JM and CV took care of the patient in the intensive care unit. All authors read and approved the final manuscript.

## Consent

We have obtained written, informed consent from the patient for open access publication of this case report and accompanying image. A copy of the written consent is available for review by the Editor-in-chief of this journal.
